# IDENTIFYING SOCIAL MEDIA RESOURCES AVAILABLE TO CAREGIVERS OF ALS PATIENTS

**DOI:** 10.1093/geroni/igz038.3341

**Published:** 2019-11-08

**Authors:** Sandra Sanchez-Reilly, Sireesha Vutukuri, Paula Reilly, Isabela S Ross, Andrea Mejia, Jeanette Ross

**Affiliations:** 1 South Texas Veterans Health Care System, San Antonio, Texas, United States; 2 The University of Texas Health Science Center at San Antonio, San Antonio, Texas, United States; 3 Health Careers High School, San Antonio, Texas, United States; 4 International School of the Americas, San Antonio, Texas, United States; 5 Colegio Mayor de Nuestra Senora del Rosario School of Medicine, Bogota, DE, Colombia; 6 University of Texas Health Science Center San Antonio, San Antonio, Texas, United States

## Abstract

Background: Amyotrophic Lateral Sclerosis (ALS) is a progressive neurodegenerative disorder that causes muscle weakness, disability, and death, with survival of 3-5 years and affecting all populations including Hispanics. ALS patients are mostly cared for by family caregivers (FCG) who experience burden, psychological distress and impaired quality-of-life. Thus, FCG may not leave their homes to access resources. Social media might be a way to accessing support, but little is known about quality/quantity of AL- FCG resources. Research Objectives: To identify and categorize types of bilingual resources available to ALS-FCG in Facebook. Methods: We used Facebook both groups (public and closed groups) and pages and collected numbers-of-likes, organization type and resource provided. Bilingual search terms (Spanish) included ALS/ALS-caregiver. Results: 24 pages/31 groups in English vs. 37 pages/29 groups in Spanish. English resources had more likes/members (3510/790 vs.410/95; p<0.001) and were more active (60+ posts in recent month). Community resources and provision of social support were similarly shared through groups in both languages; advocacy was more prominent among Spanish groups (69%vs.39%, p<0.001). English pages significantly provided more social support (96% vs.65%, p<0.001), more caregiver skills (67%vs.49%, p<0.001), inspirational roles (88% vs.59%, p<0.001). Spanish pages and groups were better in providing news/research to FCG (59%vs.16%, p<0.001). Implications for research, policy or practice: Facebook provides numerous useful bilingual resources to ALS-FCG. FCG may receive social support, inspiration, training, news/research and advocacy. Further interventions need to actively improve the role of social-media in supporting bilingual ALS FCG and use it as a platform of dissemination.

